# Chemical Analysis and Flavor Distribution of Electronic Cigarettes in Australian Schools

**DOI:** 10.1093/ntr/ntae262

**Published:** 2024-11-12

**Authors:** Caitlin Jenkins, Fraser Powrie, Celine Kelso, Jody Morgan

**Affiliations:** Molecular Horizons, University of Wollongong, Wollongong, New South Wales, Australia; School of Chemistry and Molecular Bioscience, University of Wollongong, Wollongong, New South Wales, Australia; NSW Ministry of Health, Centre for Population Health, Tobacco and e-Cigarette Control Unit, Sydney, New South Wales, Australia; Molecular Horizons, University of Wollongong, Wollongong, New South Wales, Australia; School of Chemistry and Molecular Bioscience, University of Wollongong, Wollongong, New South Wales, Australia; Molecular Horizons, University of Wollongong, Wollongong, New South Wales, Australia; School of Chemistry and Molecular Bioscience, University of Wollongong, Wollongong, New South Wales, Australia

## Abstract

**Introduction:**

Adolescent usage of electronic cigarettes has increased globally. Inconsistent, or absent, labeling of nicotine and other ingredients requires chemical analysis to accurately determine the chemical composition of these products.

**Aims and Methods:**

Electronic cigarettes confiscated from public and private high school students (*N* = 598) were provided for analysis from three regions in New South Wales, Australia. The products were examined for brand, model, and flavor and a subset was further analyzed for chemical composition (*n* = 410) quantifying nicotine, synthetic cooling agents, flavoring chemicals, and prohibited ingredients by gas chromatography–mass spectrometry (GC–MS).

**Results:**

The majority of samples provided were fruit-flavored disposable e-cigarettes across three main brands (IGET, HQD, and Gunnpod). Nicotine was quantified in 97.3% of disposable samples with an average concentration of 40.0 mg/mL, while one refill e-liquid was found to contain nicotine at a low concentration. Almost all samples contained the coolant WS-23 in relatively high concentrations compared to other flavoring chemicals present. Chemicals prohibited under the TGO110 (Australian e-cigarette product standard) were identified in 3.4% of the samples which were chemically analyzed. This included the presence of ethylene glycol in moderately high concentrations (up to 13.2 mg/mL).

**Conclusions:**

Australian students’ preferences for fruity, disposable e-cigarettes were identified regardless of region with the vast majority containing high concentrations of nicotine. WS-23 was found in most disposable e-cigarettes, potentially to reduce throat irritation from nicotine and other flavoring chemicals. The inhalational safety of the samples is of concern due to health risks associated with detected prohibited compounds, particularly ethylene glycol.

**Implications:**

This is the first study to quantify nicotine, coolants, and flavoring chemicals in e-cigarette products seized from Australian high school students and has significant implications for future policy development. Students appear to be almost exclusively using disposable e-cigarettes with high nicotine concentrations and predominately fruity flavors. WS-23 may potentially be added to disposable e-cigarettes to facilitate the uptake of these products by adolescents unaccustomed to the throat irritation from nicotine and intense flavors. The e-cigarette coils were found to have degraded over time, potentially affecting the composition of the aerosol and leaching of metals.

## Introduction

Adolescent usage of electronic cigarettes (e-cigarettes or vapes) is becoming increasingly popular in many countries.^1,2^ The latest data from 2022 to 2023 has reported that 29.9% of Australian high school students had ever used an e-cigarette, more than doubling rates from 2017, with 3% reporting daily vaping in the past month.^3^

E-cigarettes were originally designed as an alternative type of nicotine delivery system to combustible cigarettes.^4^ They contain a liquid, referred to as an e-liquid, that is vaporized to produce an aerosol for inhalation by the user. The e-liquid generally contains the carrier fluids propylene glycol (PG) and vegetable glycerin (VG), nicotine, flavoring chemicals, and occasionally synthetic cooling agents (referred to as coolants).^5–7^ There is little published data on the inhalational safety of the flavoring chemicals and synthetic coolants, and the long-term effects of e-cigarette usage are relatively unknown. Reactions between some flavoring chemicals and the carrier fluids have been observed to form acetals in situ in e-liquids, the toxicological properties of which are similarly understudied.^8–10^

In Australia, nicotine-containing vaping products are a Schedule 4 medicine, requiring individuals to obtain a prescription from a medical practitioner for purchase.^11^ From July 2024, reforms to Australian legislation limited the supply of all e-cigarettes to pharmacies, with the prescription requirement removed for products with less than 20 mg/mL of nicotine from October 2024.^12^ The standard for nicotine vaping products (TGO110)^13^ establishes guidelines for the contents of nicotine-containing e-cigarette products and their labeling. There are eight ingredients (2,3-pentanedione, acetoin, benzaldehyde, cinnamaldehyde, diacetyl, ethylene glycol, diethylene glycol, and vitamin E acetate) that have been prohibited from inclusion in e-cigarette products due to associated health risks.^13^ Nicotine is the only permitted active ingredient, the presence of which must be clearly labeled with the concentration of nicotine in the product within 10% of the stated concentration.^13^

Prior to 2024 Australian legislative reforms, e-cigarette products could not be purchased by anyone under 18 years of age in all states or territories, regardless of whether these products contain nicotine.^14^ Despite this, Australian adolescents are accessing e-cigarette products, reportedly from friends, online, and instore, with relative ease.^15,16^ An audit of online retailers that advertised delivery to the Australian city of Perth found only half required age verification for purchase, often in the form of a simple age confirmation button or input of a date of birth, which was easily falsified.^17^ The 2024 legislative changes resulted in the closure of all businesses legally selling nicotine-free e-cigarette products, although it is unclear how this will affect the Australian illicit market.

Trends in e-cigarette products can be observed to change as new device types are introduced, evidenced by the introduction of JUUL pods in 2015^18^ and Puff Bar disposable devices in 2019^19^ in the United States and their subsequent surge in popularity. Along with device trends, nicotine strength has increased in the United States for disposable e-cigarettes compared to refillable e-liquids since 2017,^20^ with recent research showing a similar trend for disposable devices on the Australian market.^5^

Several recent surveys of Australian adolescents have provided insights into the current landscape of e-cigarettes amongst this population.^15,16,21,22^ Disposable e-cigarette devices are reportedly the most popular type of e-cigarette product obtained with fruity flavors being preferred.^15,16,21^ Notably, adolescents have expressed a preference for nicotine-containing e-cigarettes, although a quarter of adolescents are unsure of the nicotine strength of their products.^21^ E-cigarettes are becoming a problem in schools with surveyed students commonly reporting observing other students’ e-cigarette use in school bathrooms and locker rooms.^15^

To date, only two studies have analyzed the chemical composition of e-cigarette products confiscated from school students. Shamout et al.^7^ quantified nicotine, PG and VG in JUUL pod devices (*n* = 26) from United States high school students while Frinculescu et al.^23^ quantified two illicit drugs and qualitatively detected nicotine, solvents, and flavoring chemicals in e-cigarette products (*n* = 70) from UK students aged 16–18. This study is the first, globally, to focus on the analysis of disposable e-cigarette products seized from school-aged students and perform quantitative analysis of flavoring chemicals along with nicotine. This paper will examine the distribution of brands, models, and flavors of a convenience sample of devices confiscated from Australian students and report the chemical composition for a large subset of the samples including nicotine content; compliance to TGO110; carrier fluid composition; and flavoring chemicals present.

## Materials and Methods

### Materials

Standards of carrier fluids (2), nicotine, synthetic coolants (2), flavors (39), internal standard (1), additional bioactives (2), and prohibited ingredients as per TGO110 (8) were purchased for chemical analysis. All analytical standards were of at least 98% purity. A list of all purchased standards and their origins is provided in Table S1.

### Study Samples

The current study investigated the brand, model, and flavor of a convenience sample of disposable e-cigarette devices (*n* = 593, from 18 high schools) and refill e-liquids (*n* = 5) confiscated from Australian high school students in New South Wales (NSW) from four distinct geographical regions: Western Sydney, Northern Sydney, Illawarra Shoalhaven, and Central Coast. For details on the origin and collection date of the samples included in this study see Table S2.

### Device Flavor Classification

All samples (*N* = 598) were visually examined and the brand, model, and flavor were identified from the exterior labeling of the disposable devices and the two bottled samples or, where more information was required, a search of online retailers was conducted to identify the sample. Any samples that did not have any clear indication of their brand, model, and/or flavor were recorded as unknown. The flavors were classified into seven categories (beverage, candy, cooling, dessert, fruit, tobacco, and other) based on the flavor categories established by Krüsemann et al.^24^ For flavors that would fit into two or more of the established flavor categories, classification was based on the highest priority flavor category (see Figure S1).

### Chemical Analysis

Chemical analysis was carried out using gas chromatography–mass spectrometry (GC–MS) on a convenience sample (*n* = 410). Quantification of nicotine, flavoring chemicals, and coolant molecules within e-liquids and disposables was achieved through the creation of internal calibration curves using quinoline as the internal standard (25 μg/mL). Instrument conditions have been previously reported^25^ with additional information provided in Text S1. Due to an extended time between analyses, two calibration sets (referred to as Set A and Set B) were produced for accurate quantitation of all samples (see Table S3). Acetal peaks were identified by comparing their retention time and mass spectrum to an in-house database created from the analysis of acetals previously synthesized in our laboratory.^8^ All samples were prepared and analyzed in triplicate. An example of a typical chromatogram has been provided in Figure S2. Five flavor chemicals were confirmed but not quantified through a comparison of retention time and mass spectrum to purchased standards (labeled as either detected or not detected). Any unknown compound peaks were tentatively identified using the National Institute of Standards and Technology (NIST17) library for spectral matches at 85% or above. A list of tentative matches is provided in Table S4. As these matches were not confirmed against standards, they will not be discussed further.

## Results

### Refill e-Liquids

Only four refillable devices were confiscated as part of this study, three of these containing an unidentified e-liquid (SCH-007, SCH-184, and SCH-185) and the fourth in a case with two labeled e-liquid bottles (SCH-029 and SCH-030). Identification of the brand and flavor of the samples was only possible for the two refill e-liquids provided in bottles which were classified as fruit and beverage-flavored. Analysis of the chemical content of the refill e-liquids was conducted for SCH-007, SCH-029, and SCH-030. Only SCH-007 contained nicotine which was detected at a low concentration (2.27 ± 0.07 mg/mL) with no benzoic acid. This was also the only refill e-liquid that contained a cooling agent (WS-23, <LOQ). One flavoring molecule (vanillin) was detected in SCH-007 while two were detected in SCH-029 (1,3-diacetin and ethyl vanillin) all in concentrations <3 mg/mL. SCH-030 contained no detected flavoring molecules. PG and VG were detected as the carrier fluids in all refill liquids.

All results below relate to the disposable e-cigarette samples.

### Brand and Model

**Figure 1.**
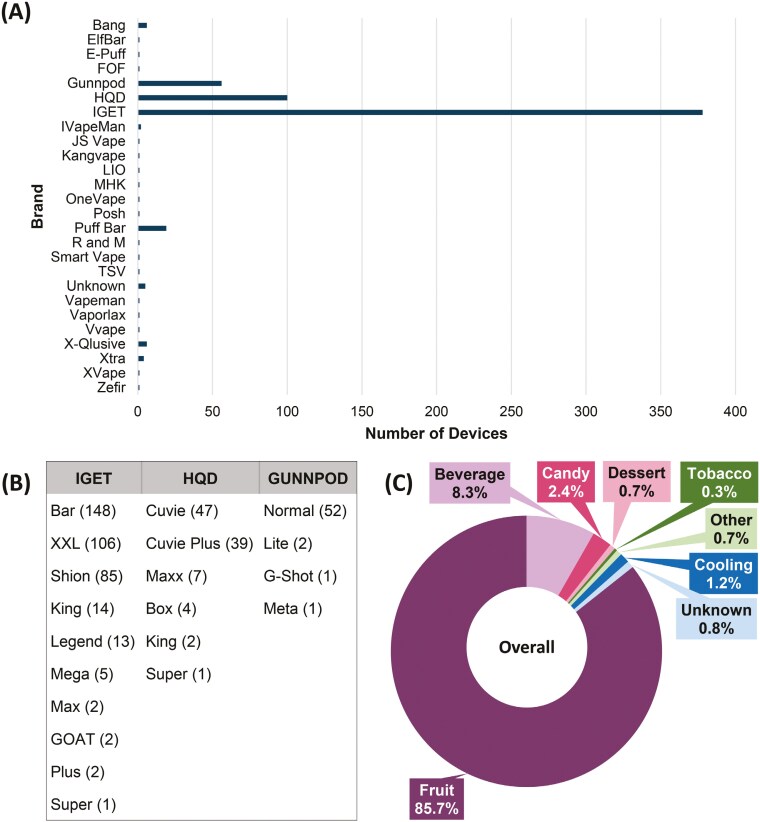
Distribution of (A) brands for disposable e-cigarettes (*n* = 593); (B) model distribution for the top three brands (*n* = 534); and (C) flavor categories from e-cigarette labeling (*n* = 593). Full details of brand, model, and flavors are provided in the Table S5.

Of the 593 disposable devices analyzed, 47 different models across 26 brands were represented (models of the same name but different brands were counted separately). The number of brands (*n* = 5) in the Northern Sydney dataset was lower than in the other regions despite a larger number of samples (*n* = 239). Across all four analyzed regions (Western Sydney, Northern Sydney, Illawarra Shoalhaven, and Central Coast) the same trend was observed in brand prevalence for the disposable devices; with the three most popular brands being IGET (63.7%), HQD (16.9%), and Gunnpod (9.4%) (Figure 1A). The fourth most prevalent brand overall, PuffBar (3.2%), was found in all regions except Northern Sydney. All other brands combined accounted for <7% of all samples and, excluding Bang, were different for each region. Of interest, one device (SCH-032, brand: Zefir) was advertised online as a pharmacy-only product.^26^ The bar model of the IGET brand was the most commonly identified device (Figure 1B), though it is worth noting that this trend is not reflected in the individual datasets. IGET Bar was the most common device for Western Sydney and Central Coast but the second most popular for Northern Sydney and Illawarra Shoalhaven. The distribution of model prevalence for HQD and Gunnpod devices was similar between all regions and is reflected in the overall data (Figure 1B). Only nine samples were provided in the original packaging, all IGET Bars from the Central Coast, and were seized from a student intending to sell on school grounds. Neither the word nicotine nor any associated concentrations were present on the packaging of these nine samples.

### Labeled Flavor

A total of 132 unique flavors were recorded with fruity flavors being the most prevalent accounting for 85.7% of all products (Figure 1C). Tobacco flavors were the least prevalent and were only present in the Northern Sydney and Central Coast samples. Dessert flavors were only identified in the Western Sydney samples. Beverage flavors were present in all of the datasets but were higher in the Illawarra Shoalhaven samples (17.9%). Almost half (*n* = 267, 45.0%) of all flavors contained a cooling component in their flavor name (eg “ice,” “iced,” “frozen,” or “cool”). Only seven samples (1.2%) were classified in the cooling category based on the flavor wheel developed by Krüsemann et al.^24^ and the flavor priority list (see Figure S1 and Table S5, flavors such as “Apple Ice” were classified as fruit according to the priority list).

### Nicotine

**Figure 2.**
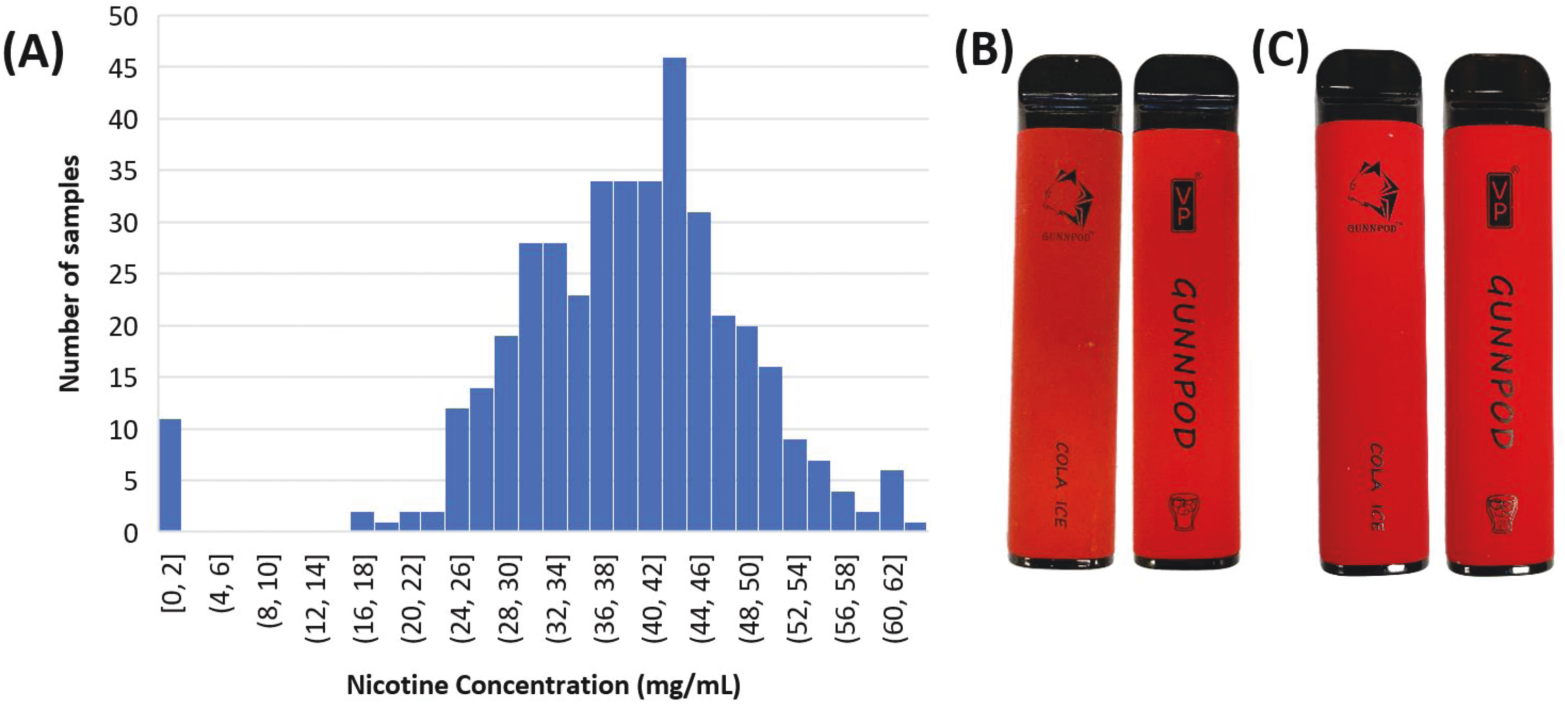
(A) Distribution of nicotine concentration in disposable e-cigarettes (*n* = 407) and comparison of (B) nicotine-free (MOH-649) and (C) nicotine-containing (MOH-300) devices of the same brand, model and flavor. Detailed measurements for each analyzed sample are provided in the Table S6 along with a short list describing the samples that were found to be nicotine-free (Table S7).

Nicotine was detected in 396 disposable e-cigarettes (*n* = 407, 97.3%) with concentrations ranging between 16.5 ± 0.4 mg/mL (SCH-006) and 63 ± 2 mg/mL (MOH-328) and a mean of 40.0 mg/mL (Table 1). Figure 2A shows the distribution of nicotine concentrations for all analyzed e-cigarette samples. Nicotine was confirmed as the nicotine benzoate salt in all samples except SCH-039 (99.75%). A total of 11 disposable e-cigarette samples (2.7%) were identified as being nicotine-free. Of the 11 nicotine-free samples, six were identical in brand, model, flavor, and packaging to at least one other nicotine-containing sample in this study (for an example see Figure 2, B and C). Two of these six devices appeared to have been opened previously, indicating that the contents of the device, including the e-liquid, may have been tampered with. Comparison of nicotine concentrations between any identical devices (brand, model, and flavor) where four or more were present generally exhibited large variations in nicotine concentration (>5 mg/mL). Of all the samples tested, only 21 devices (3.5%, 19 Puff Bar, 1 MHK, and 1 Xtra) were labeled with any type of concentration, shown as “5%”, all without the inclusion of the word nicotine. Eighteen of these “5%” labeled products were analyzed for content and none of them met the ±10% criteria outlined in the TGO110.

**Table 1.** Summary of Analyzed Disposable e-Cigarettes (*n* = 407) for Detected Compounds

Chemical name (and its associate flavor^a^)	Number of samples containing this chemical	% of samples containing this chemical	Number of samples with quantifiable concentration	Average concentration (mg/ml)	Minimum concentration (mg/mL)	Maximum concentration (mg/mL)
Nicotine						
Nicotine	396	97.3	396	40.0	16.5 ± 0.4	63 ± 2
Flavor chemicals						
*p*-Anisaldehyde (“butter almond”)	10	2.5	6	0.37	0.18 ± 0.02	0.67 ± 0.02
Benzyl alcohol (“cherry, floral”)	136	33.4	49	1.26	0.406 ± 0.004	6.8 ± 0.1
Benzyl benzoate (“faintly fruity”)	25	6.1	11	0.34	0.069 ± 0.002	0.75 ± 0.01
Butanoic acid (“pungent, acidic”)	11	2.7	2	1.41	1.03 ± 0.05	1.78 ± 0.05
δ-Decalactone (“coconut, creamy”)	6	1.5	0	–	–	–
γ-Decalactone (“fruity, peach”)	153	37.6	73	0.28	0.0612 ± 0.0009	0.93 ± 0.01
1,2-Diacetin (“fatty, buttery”)	195	47.9	182	4.40	0.048 ± 0.005	23.9 ± 0.6
1,3-Diacetin (“fatty, buttery”)	225	55.3	192	11.8	0.090 ± 0.005	101 ± 4
Diethyl succinate (“winey”)	7	1.7	1	0.18	0.176 ± 0.004	0.176 ± 0.004
Ethyl butanoate (“pineapple”)	7	1.7	1	0.86	–	–
Ethyl maltol (“sweet, caramel”)	173	42.5	78	3.25	0.718 ± 0.009	18.1 ± 0.4
Ethyl vanillin (“vanilla”)	30	7.4	16	1.00	0.29 ± 0.01	2.06 ± 0.04
*cis*-3-Hexene-1-ol (“green, grassy”)	114	28.0	68	0.42	0.171 ± 0.004	1.51 ± 0.05
Isoamyl acetate (“banana”)	4	1.0	2	0.47	0.46 ± 0.01	0.48 ± 0.04
Isoamyl isovalerate (“fruity, apple”)	1	0.2	0	–	–	–
Maltol (“sweet, candy”)	11	2.7	1	4.77	–	–
Menthol (“mint”)	100	24.6	51	0.74	0.40 ± 0.02	1.81 ± 0.05
Methyl cinnamate (“cinnamon”)	84	20.6	33	0.21	0.097 ± 0.005	0.66 ± 0.01
3-Methyl-1,2-cyclopentanedione (“caramel, maple”)	2	0.5	1	0.60	–	–
γ-Nonalactone (“sweet, vanilla”)	12	2.9	12	5.09	4.0 ± 0.2	6.1 ± 0.5
Piperonal (“cherry, spicy”)	3	0.7	0	–	–	–
Sulfurol (“meaty, roasted, nutty”)	6	1.5	1	2.54	–	–
γ-Undecalactone (“fatty, creamy”)	98	24.1	44	0.22	0.078 ± 0.009	0.74 ± 0.05
Vanillin (“vanilla”)	175	43.0	110	1.33	0.23 ± 0.02	8.2 ± 0.3
Coolants						
WS-23	405	99.5	403	14.2	0.278 ± 0.006	32.5 ± 0.7
WS-3	63	15.5	60	1.15	0.250 ± 0.007	2.70 ± 0.04
Prohibited compounds						
Acetoin (“buttery”)	2	0.5	0	–	–	–
Benzaldehyde (“almond”)	3	0.7	2	0.31	0.30 ± 0.01	0.310 ± 0.009
Cinnamaldehyde (“cinnamon”)	5	1.2	1	2.23	–	–
Ethylene glycol (odorless, “sweet tasting”)	4	1.0	3	9.22	3.35 ± 0.08	13.2 ± 0.2

^a^Chemical flavors associated with this chemical as identified from the Good Scents database^27^. Flavor chemicals which were not identified in any samples: 4-acetylanisole, 2-acetylpyridine, p-anisyl alcohol, p-dimethoxybenzene, ethyl hexanoate, furfural, guaiacol, 1-methylnaphthalene, valeraldehyde, veratraldehyde. Prohibited compounds which were not identified in any samples: diacetyl, diethylene glycol, 2,3-pentanedione, and vitamin E acetate.

### Other Bioactive Compounds

Cannabidiol (CBD) and Δ-9-tetrahydrocannabinol (THC) were included as standards for the chemical quantification of all analyzed samples. Neither of these bioactive compounds was detected in any samples in this dataset (see Table S6).

### Flavor Chemicals

There was a large variation observed in the frequency of appearance and concentration of flavors detected among the samples (Table 1). The most commonly detected flavoring chemicals were 1,3-diacetin (55.3%), 1,2-diacetin (47.9%), vanillin (43.0%), and ethyl maltol (42.5%). The most concentrated flavoring chemical was 1,3-diacetin with concentrations as high as 101 ± 4 mg/mL (MOH-269). With the exception of 1,3-diacetin, flavor concentrations were, on average, present in all samples either in low concentrations (<2 mg/mL) or moderate concentrations (2–6 mg/mL). Raspberry ketone, triethyl citrate, and ethyl-3-methyl-3-phenylglycidate were detected and confirmed to be present but not quantified in 82 (20.1%), 40 (9.8%), and 32 (7.9%) samples respectively. The composition of flavoring chemicals in identical devices (brand, model, and flavor) where four or more were present was different; however, large variations (>5 mg/mL) were generally only observed for flavoring chemicals in high concentrations. The large variation value was selected as an appropriate range due to the large spread in concentrations for different flavoring molecules. Concentration measurements for each sample analyzed are provided in the Table S8. PG and VG were detected as the carrier fluids in every sample with an average ratio of 30%PG/70%VG.

### Coolants

WS-23 was detected in 405 of the samples (99.5%), of which 63 also contained WS-3, with average concentrations of 14.20 mg/mL and 1.15 mg/mL, respectively (Table 1, with full details provided in Table S8). There was no correlation between the inclusion of a cooling component in the flavor name and the presence of a coolant. WS-23 was, on average, the second most concentrated ingredient in disposable e-cigarettes and more concentrated than most flavoring chemicals. Comparison of WS-23 concentrations between any identical devices (brand, model, and flavor) where four or more were present generally exhibited large variations (>5 mg/mL).

### Prohibited Compounds

Four compounds prohibited by the TGO110 were detected in disposable e-cigarettes in this study. A total of 14 samples (3.4%) contained one prohibited compound, no samples contained more than one prohibited substance. The detected prohibited compounds were acetoin (*n* = 2), benzaldehyde (*n* = 3), cinnamaldehyde (*n* = 5), and ethylene glycol (*n* = 4) (Table 1, with full details provided in Table S9 and S10). Two of these samples (MOH-304 and MOH-649) did not contain any nicotine and therefore their content was not required to abide by TGO110.

### Acetals

The PG acetal of p-anisaldehyde and the PG and VG acetals of vanillin and benzaldehyde were detected in this study. Formation of acetals was only detected in six samples (1.5%) (see Table S11), all of which were shown to also contain the original flavoring molecule.

### Coil Degradation

**Figure 3.**
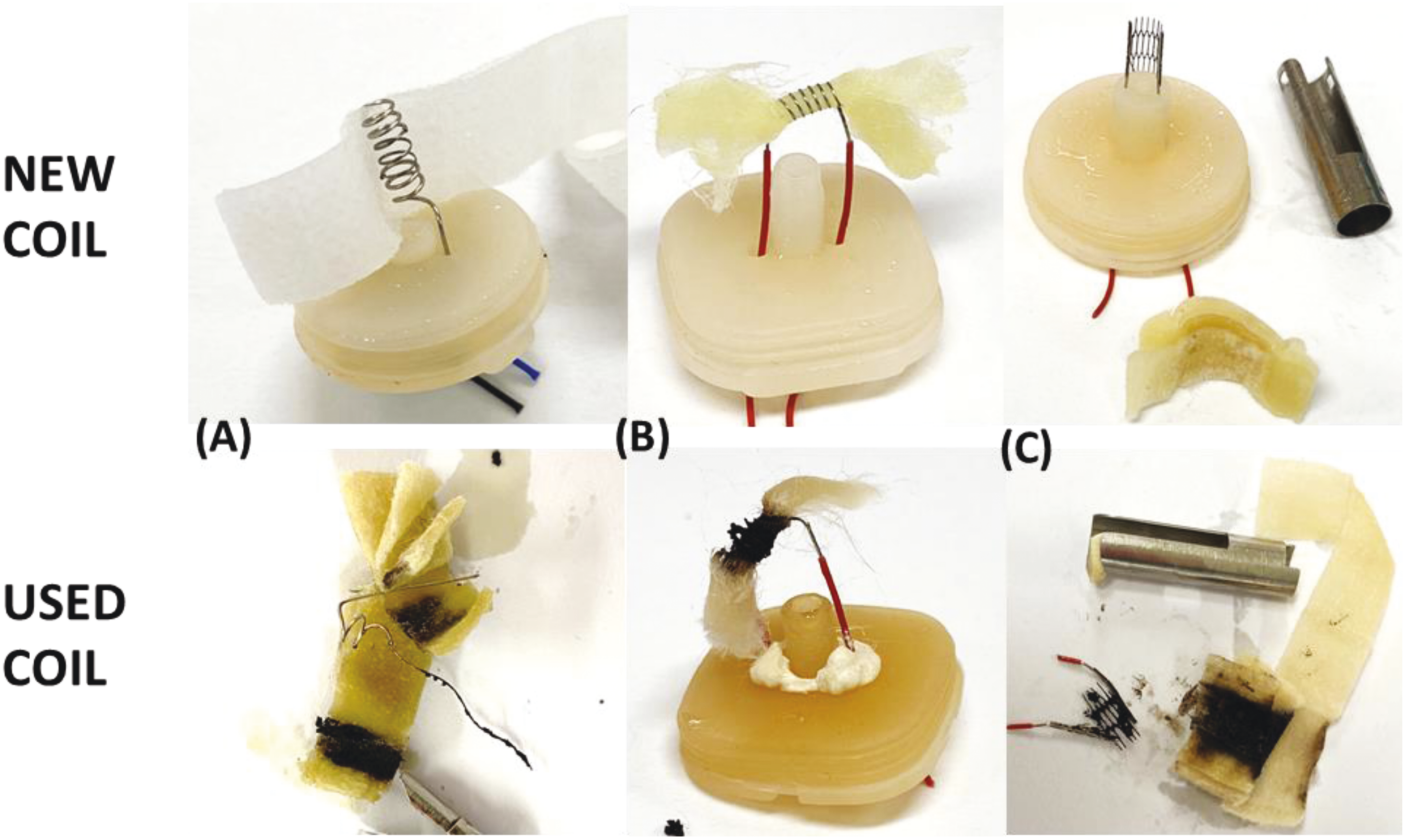
Comparison of new (top) and used (bottom) metal heating coils of types (A) vertical coil in a new device and MOH-646, (B) horizontal coil in a new device and SCH-022 and (C) mesh coil in a new device and MOH-633.

Coils in disposable e-cigarettes were observed in various stages of degradation via the blackening of the metal coil and scorching of the surrounding fabric (Figure 3). The degree of charring was considerably different between samples with some showing little to no blackening and some where the coil was indistinguishable from the scorched fabric (Figure 3). A greater degree of charring was generally found in devices with lower volumes of e-liquid remaining or tampered devices.

## Discussion

Australian high school students were found to primarily use disposable e-cigarette products with high concentrations of nicotine regardless of region or type of school (public vs. private), some of which contained potentially harmful compounds. These results are consistent with several recent surveys of Australian adolescents where they expressed preferences for nicotine-containing, fruit-flavored disposable e-cigarettes.^15,16,21^ High school students in NSW, Australia are generally between the ages of 12 and 18, with almost all students below the minimum legal age to purchase e-cigarettes.

Refillable e-cigarette devices accounted for <1% of samples in this study, suggesting that these types of e-cigarette products are uncommon among high school students in NSW. The small number of refill e-liquids analyzed were more likely to be nicotine-free and coolant-free compared to the disposable devices. While the sample size for refillable e-liquid analysis was small due to this study relying on a convenience sample, these results are consistent with our previous research comparing Australian refillable e-liquids and disposable e-liquids.^5^ As the vast majority of students use disposable e-cigarettes, the prohibition of these devices in the 2024 Australian legislative reforms may have a significant effect on youth vaping.

The similarity between brand and model distributions in the analyzed regions may suggest broader trends across Australia, although these are inconsistent with results reported in recent surveys.^15^ PuffBar products, which were identified by Pettigrew et al.^15^ as the most popular brand among Australian 15–21 year olds, only accounted for 3.2% of the samples confiscated from high school students in this study. Differences between this study and Pettigrew et al.^15^ could reflect broader variations in the Australian market following the regulatory changes in October 2021, when the survey was conducted, as the samples in this study were received at least 1 year after this date. Additionally, JUUL, which was reported by Pettigrew et al.^15^ as the third most common brand among this age group was not present among the confiscated samples. JUUL is known to be a common brand in the United States^18^ and the term “JUULing” is sometimes used interchangeably with “vaping.”^28,29^ It is possible that students are self-reporting the term “JUUL” to describe any disposable vaping device rather than identifying a specific brand.

Consistent with the literature,^15,21,30^ fruity flavors were the most popular while tobacco-flavored devices were uncommon. The large variation in the number and concentration of flavoring chemicals in each sample and frequency of appearance reflects the large range of unique flavors analyzed. Two of the four most commonly detected flavors were sweet flavors,^27^ vanillin and ethyl maltol, reflecting the overwhelming number of fruity and sweet-flavored samples in this study. An online search (August 2023) of the two most popular models from this study, the IGET Bar and XXL, identified a wide range of fruity flavors available for purchase but no tobacco flavors, possibly as a result of user preferences.^31,32^

Chemical analysis was only conducted on one tobacco-flavored device which was found to contain mainly “sweet” flavoring chemicals (3-methyl-1,2-cyclopentanedione, ethyl maltol, vanillin, and ethyl vanillin) and the “meaty” flavoring chemical sulfurol.^27^ These sweet flavoring chemicals were observed in concentrations similar to or exceeding that of the fruit-flavored disposable e-liquids. Recommendations to restrict e-cigarette flavors, such as reforms in Australia,^12^ often recommend allowing tobacco to remain available and some research has found that tobacco flavors may be less appealing.^30^ These flavor restrictions would only apply to legal e-cigarette products accessed through a pharmacy in Australia. Without an indication of what chemicals comprise tobacco flavor it is unclear whether this would be effective in eliminating fruity and sweet flavors in legal e-cigarette products.

Virtually all of the disposable e-cigarettes confiscated from high school-aged students contained nicotine in salt form (97.1% nicotine benzoate) at relatively high concentrations. Conversion of nicotine to its salt form via the addition of acid reduces the throat harshness associated with its inhalation and allows for higher concentrations of nicotine to be tolerated.^33^ Additionally, the coolant WS-23 was detected in >99% of the disposables analyzed at concentrations higher than most of the other flavoring chemicals present. The only two disposable devices that did not contain WS-23 were a Watermelon Mint Ice-flavored device, which contained menthol, and a Classic Tobacco-flavored device. WS-23 may be added to disposable e-cigarettes to further reduce the throat irritation associated with high nicotine concentrations and intense flavors among naïve users.^5^ A recent survey suggested that more than half of adolescents who reported e-cigarette ever-use had never previously smoked and would therefore be unaware and unaccustomed to the throat irritation.^16^ The high concentrations of WS-23 observed throughout this study are noteworthy as previous research has found comparable concentrations to be unsafe based on in vitro studies and may adversely impact the inhalational safety of these products.^34,35^

Current Australian product guidelines for nicotine vaping products (TGO110) require the labeling of nicotine, its concentration, and warning statements on the packaging.^13^ It is a requirement that the nicotine concentration is within 10% of the labeled concentration.^13^ While no details were provided about the origin of these samples (how and where these products were purchased or obtained), it cannot be excluded that these devices were legally obtained, therefore all samples containing nicotine were compared against the TGO110 requirements to assess compliance. The vast majority of samples were not labeled and of the small number of samples that did include a nicotine concentration, the actual concentration was outside 10% of the stated concentration for all samples chemically analyzed.

An additional requirement of the TGO110 is the prohibition of eight specific chemicals that have been banned in nicotine-containing e-cigarettes due to health risks.^13^ Four prohibited compounds (acetoin, benzaldehyde, cinnamaldehyde, and ethylene glycol) were detected in 14 of the disposable e-cigarettes. All four of these compounds were prohibited due to their potential inhalational health risks; including possible lung damage, respiratory failure, cytotoxicity, and depression of the central nervous system.^13^ Of particular concern, ethylene glycol was found in moderate to high concentrations in two samples(11.1 ± 0.2 mg/mL in MOH-325 and 13.2 ± 0.2 mg/mL in SCH-070). Ethylene glycol has been found to cause respiratory irritation and is a toxicological hazard.^36,37^ While ethylene glycol has been observed in e-cigarette products previously,^37^ this is the first study to identify and quantify it in Australian e-cigarette products. It is worth noting that 2 of the 14 samples that contained prohibited compounds did not contain nicotine and consequently, their contents were not required to abide by the Australian legislation at the time of the study (2022–2023) which applied only to nicotine vaping products.

Nine disposable samples from the Central Coast region were unused and in original packaging when confiscated from a student who was intending to sell them. These samples followed the trends observed throughout this study being fruit-flavored IGET Bars. All of these unopened devices contained nicotine with no mention of nicotine on the packaging or appropriate warning messages, which is reflected in results published previously by our research group.^25^ This supports previous findings that some adolescents are sourcing their nicotine e-cigarettes from a friend or associate and provides evidence that this is occurring within school cohorts.^15,16,21^ Moreover, reports that a quarter of adolescents are unaware of the nicotine concentration of their e-cigarette products is likely due to a lack of information on the packaging of these devices.^21^

The samples were provided at different stages of use (new vs. empty) which prevented the chemical analysis of some samples due to low e-liquid yield and may have affected the composition of the collected e-liquids. Volatile flavoring molecules were detected in low concentrations in some identical devices (<2 mg/mL) and were absent in others. This is possibly due to the preferential vaporization of these compounds resulting in low concentrations or their absence at the end of the device’s life.^5^ However, this does not necessarily account for the large differences in nicotine and WS-23 concentrations in many of the identical devices. It is more likely that this is reflective of irregularity in the manufacturing of these devices^34^ or student tampering in replacing the original e-liquid.

Many of the samples from this study had coils with differing levels of charring, occurring over time as the coil undergoes repeated heating and cooling cycles to vaporize the e-liquid. Figure 3 shows the excessive blackening on some coils and additional scorching of the surrounding fabric. The level of charring differed, likely due to the different stages of usage of the samples. Degradation of e-cigarette coils and loss of metals, possibly into the aerosol, has been found previously^38,39^ and may contribute to the observed coil blackening. This is an important issue for future research as it is unclear how this degradation may affect the composition of the aerosol and the inhalational safety of e-cigarettes. The charred coils were generally observed in samples with low e-liquid volumes remaining or evident tampering, suggesting that these devices may be used beyond the expected puff capacity of the coils leading to substantial degradation.

While a large number of samples were analyzed, this study is limited by the fact that it utilizes a convenience sample of confiscated devices and was reliant on staff members to identify what e-cigarette products look like, potentially biasing the data set towards more obvious products. The samples analyzed in this paper are only representative of the products being used by Australian adolescents. All regions of study were within NSW and therefore similar research should be conducted across Australia to confirm if the results of this study are reflected in high schools across the country. The specific dates the e-cigarettes were confiscated were not provided for the majority of the samples, preventing the analysis of broader trends over time. The e-cigarette market is rapidly changing and requires regular research to identify potential variations over time, particularly as legislative reforms are introduced.

The present study provides the first analysis of e-cigarette products confiscated from Australian students. The findings of this study indicate that Australian adolescents are using fruit-flavored disposable e-cigarette devices, most commonly the IGET Bar, that generally contain high concentrations of nicotine and WS-23. Compounds prohibited due to their associated inhalational health risks were found in 3%–4% (*n* = 14) of the chemically analyzed samples. Four of these samples were found to contain the prohibited ingredient ethylene glycol, the first quantified identification of this compound in Australian e-cigarette products. Future policy should focus on preventing adolescents from accessing disposable e-cigarettes.

## Supplementary material

Supplementary material is available at *Nicotine and Tobacco Research* online.

ntae262_suppl_Supplementary_Material

## Data Availability

All data from this project is available in the Supplementary Material.
